# Multi-channel transorbital electrical stimulation for effective stimulation of posterior retina

**DOI:** 10.1038/s41598-021-89243-y

**Published:** 2021-05-07

**Authors:** Sangjun Lee, Jimin Park, Jinuk Kwon, Dong Hwan Kim, Chang-Hwan Im

**Affiliations:** 1 Department of Electronic Engineering, Hanyang University, Seoul, Republic of Korea; 2 Center for Intelligent and Interactive Robotics, Korea Institute of Science and Technology, Seoul, Republic of Korea; 3 Department of Biomedical Engineering, Hanyang University, 222 Wangsimni-ro, Seongdong-gu, Seoul, 04763 Republic of Korea; 4 Department of HY-KIST Bioconvergence, Hanyang University, Seoul, Republic of Korea

**Keywords:** Biomedical engineering, Computational biophysics

## Abstract

Transorbital electrical stimulation (tES) has been studied as a new noninvasive method for treating intractable eye diseases by delivering weak electrical current to the eye through a pair of electrodes attached to the skin around the eye. Studies have reported that the therapeutic effect of tES is determined by the effective stimulation of retinal cells that are densely distributed in the posterior part of the retina. However, in conventional tES with a pair of electrodes, a greater portion of the electric field is delivered to the anterior part of the retina. In this study, to address this issue, a new electrode montage with multiple electrodes was proposed for the effective delivery of electric fields to the posterior retina. Electric field analysis based on the finite element method was performed with a realistic human head model, and optimal injection currents were determined using constrained convex optimization. The resultant electric field distributions showed that the proposed multi-channel tES enables a more effective stimulation of the posterior retina than the conventional tES with a pair of electrodes.

## Introduction

Noninvasive electrical stimulation of the eyes has been studied as a promising therapeutic tool to recover visual functions in patients suffering from various eye diseases^1^. There are two well-known methods that deliver the electric current to the eye noninvasively. One is transcorneal electrical stimulation that delivers the currents via a contact-lens-type electrode attached right above the cornea^2,3^. Previous studies reported that transcorneal electrical stimulation has beneficial effects on the improvement of visual functions in patients with optic neuropathy^2^ and retinitis pigmentosa (RP)^4–6^. According to the studies that used animal models with eye diseases, improvement of visual functions resulting from transcorneal electrical stimulation was closely associated with the survival of the retinal ganglion cells (RGCs) and photoreceptors preserved from the degeneration, suggesting that the neuroprotective effect on retinal cells determines the outcome of the transcorneal electrical stimulation^7^. Additionally, it was found that the increase in the survival of RGCs after transcorneal electrical stimulation is related to an increase in the insulin-like growth factor 1 (IGF-1), brain-derived neurotrophic factor (BDNF), and ciliary neurotrophic factor (CNF), which are released from Müller cells in the retina^8,9^.

The other method is transorbital electrical stimulation (tES) that delivers weak electrical current to the eye via electrodes attached to the skin around the eye. The stimulation parameters such as electrode configurations, current waveforms, injection current intensities differed among studies. Generally, square pulses in bursts with the frequency range of 5–30 Hz were applied for tES^10^. Compared to transcorneal electrical stimulation, tES is less invasive with no side effects such as dry-eye and punctate keratitis and easier to apply^11^. Repetitive tES, applied to patients with optic nerve damage, has been reported to improve visual field size, visual acuity, and detection ability^12,13^. Repetitive tES has also reportedly strengthened the alpha-band functional connectivity in patients with chronic prechiasmatic visual system damage^14^. Another study has demonstrated that the tES-treated group showed a significant improvement in visual fields and reaction times during the visual-field-related task compared to the sham stimulation group^15^. Furthermore, tES has also been effective in improving visual function in patients with RP^16^. A previous study reported that the effectiveness of tES was related to the synchronization of cortical activities after retinal cells were stimulated^10^. Another study insisted that improvement of visual functions together with changes in the spectral EEG alpha band power and connectivity in the occipital lobe after tES might be caused by a retinofugal entrainment through firing of RGCs^15^. Indeed, a previous in vivo experimental study with rats also demonstrated that electrically evoked, tES-induced responses stemmed from the retina^17^. These series of findings suggest that a stronger electric field should be delivered to the cells in the retina to increase the therapeutic effect of tES.

Generally, the conventional electrode configuration used for tES comprises two active electrodes attached to the skin near the orbital cavity and a single reference electrode placed on the occipital pole or extra-cephalic regions like the wrist and neck^10,12,18^. According to a numerical simulation study with the conventional electrode montage, most electric fields were delivered to the anterior part of the eye^15^. Therefore, the conventional tES dominantly stimulated the anterior part of the retina despite a large number of retinal cells, including RGCs and Müller cells, being densely distributed in the posterior part of the retina, particularly around the fovea^19^. Therefore, considering the abovementioned action mechanisms of both tES, a stronger electric field should be delivered to the posterior part of the retina to increase the effectiveness of tES. In the conventional tES, however, electric field delivered to the peripheral side of the retina (anterior retina) reaches an individual phosphene threshold^20^, which represents the maximally allowable injection current in tES that does not evoke phosphenes in an individual, before a sufficient amount of stimulation current is delivered to the posterior retina. Therefore, it is necessary to reduce the electric field delivered to the anterior retina in relative to that delivered to the posterior retina to maximize the overall therapeutic effects of tES.

This study proposes a novel tES montage with eight active electrodes, with the diameter of 1 cm, attached around the eye (approximately 2 cm away from the center of the cornea) and a reference electrode on the occipital pole to reduce the difference in the electric field intensities delivered to the anterior and posterior retina. In other words, the study aims to maximize the electric field delivered to the posterior retina when that delivered to the anterior retina reaches the individual phosphene threshold. As aforementioned, short duration square pulses at a specific frequency are generally employed for tES. Although the electrical conductivity values of tissues are dependent on the frequency of injected current^21–23^, we employed tissue electrical conductivity values at DC frequency and solved a quasi-static Laplace equation because the frequency range used for tES (5 – 30 Hz) was low enough for the quasi–static approximation. Indeed, it was reported that there was no difference between the electric fields calculated assuming DC and AC with a relatively high frequency (~ 1 kHz)^24^. The optimal injection currents of the active electrodes were determined to maximize the electric field delivered to the posterior retina, near the fovea, by employing a constrained convex optimization approach. The efficacy of the new stimulation conditions was evaluated by comparing it with the conventional electrode montage.

## Methods

### Construction of finite element model

**Figure 1:**
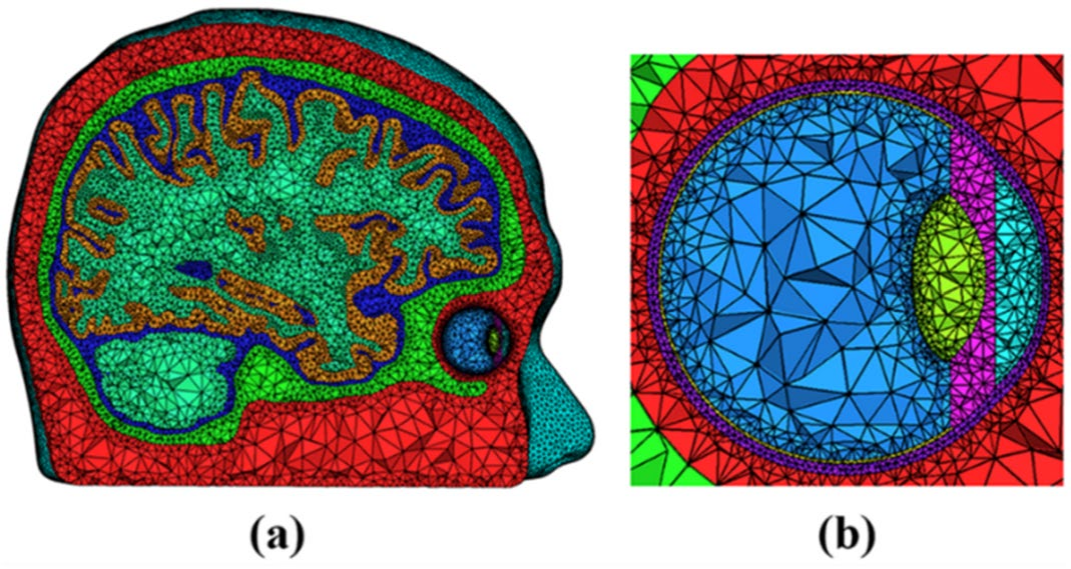
(**a**) Finite element model of the human head—cross-sectional view. (**b**) Enlarged visualization of the segmented human eye composed of tissues: sclera (purple), vitreous body (blue), retina (yellow), lens (green), ciliary body, and iris region (pink), and anterior chamber (sky blue).

**Figure 2:**
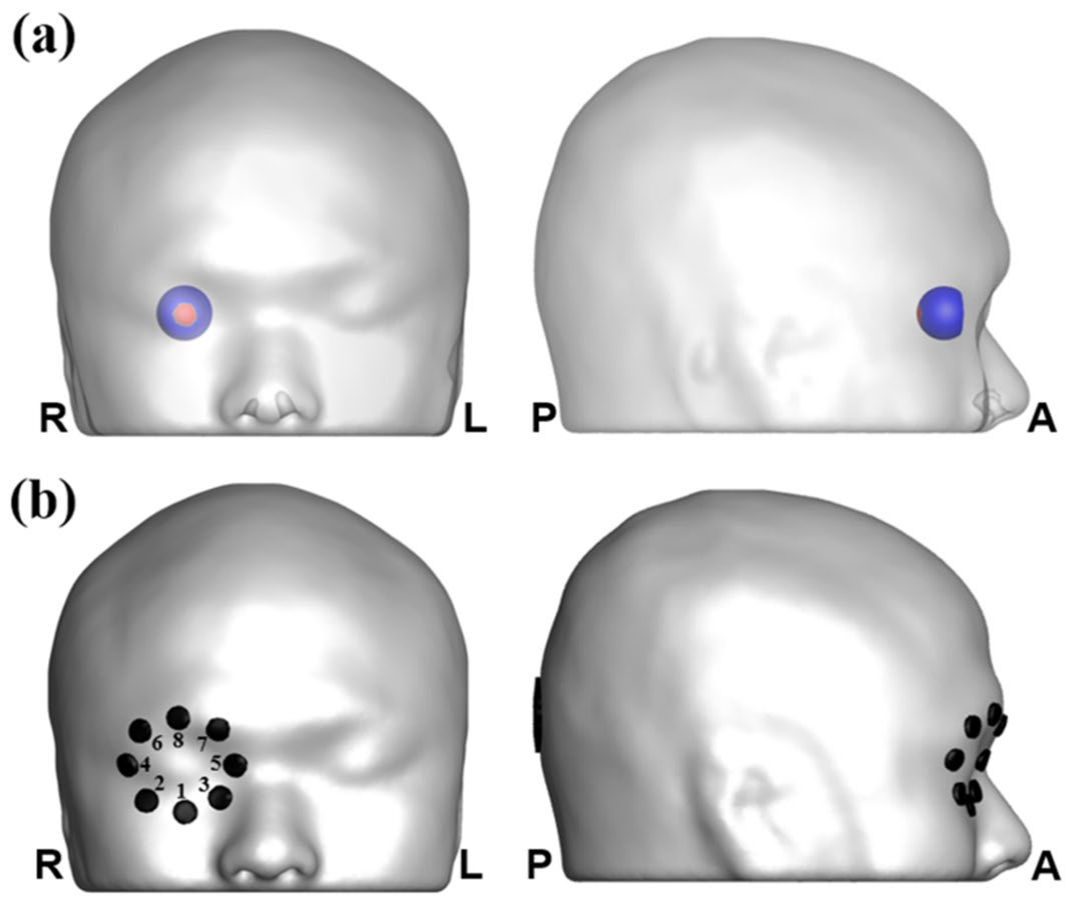
(**a**) Illustration of the whole retina (blue) and region of interest (ROI) (red). (**b**) Illustration of the suggested montage with eight active electrodes. The electrode number was listed next to the electrode.

A realistic finite element (FE) human head model was constructed using T1-weighted magnetic resonance (MR) images of a young male subject (26 years old), which were acquired from a 3 T MAGNETOM Trio scanner (Siemens, Erlangen, Germany) with a resolution of 1 × 1 × 1 mm. The subject was required to provide a written informed consent after he had been informed of the purpose of the experiment. He also agreed the publication of his head images in an online open-access publication by signing the written informed consent. The experimental protocol was approved by the Institutional Review Board (IRB) Committee of Hanyang University (HYI-17-180-5). All data acquisitions were performed in accordance with the guidelines and regulations set by the IRB of Hanyang University. The SimNIBS v2.0 was used to automatically segment head tissues, including those of the scalp, skull, cerebrospinal fluid (CSF), gray matter, and white matter^25^. The right eye was segmented into six tissues with different electrical conductivities: the sclera, vitreous body, retina, lens, ciliary body with iris, and anterior chamber, using the ANSYS v18.2 (ANSYS Inc., PA, USA) (see Fig. 1b). Detailed dimensions of the eye were determined according to a previous literature^26^. We combined the head model with the eye model to create a volumetric FE model, with the eye’s location determined based on the original MR images (see Fig. 1a). The surface of the posterior retina, the target area of tES in this study, was set as a region of interest (ROI), as shown in Fig. 2a. Then, an in-house script was coded to correct segmentation errors and improve the quality of tetrahedral elements by removing isolated nodes and self-intersecting elements using Matlab 2018a (Mathworks, Natick, MA, USA). A prior study details the correction processes^27^. The final FE head model consisted of 604,554 nodes and 3,666,722 tetrahedral elements. This study proposed a new tES electrode montage consisting of eight active electrodes, attached to the facial surface approximately 2 cm away from the center of the cornea, and one reference electrode, placed at Oz according to the international 10–10 EEG electrode system (see Fig. 2b). We assumed circular cylindrical-shape electrodes that can be readily attached on the facial skin or scalp surface with adhesive sticker electrodes or saline-soaked sponge electrodes that have been widely used for multi-channel transcranial electrical stimulation systems^28^. The active and reference electrodes were modeled as thin cylinders with a thickness of 2.5 mm, the diameters of which were set to 1 cm and 3.4 cm, respectively. We did not consider the rotation of the eyeball because human subjects are usually asked to close their eyes during the entire stimulation session in practical tES applications^12^.

### Determination of optimal injection currents

The optimization approach used in a previous transcranial direct current stimulation (tDCS) study^29^ was employed to determine each electrode’s optimal injection currents capable of delivering a maximum electric field to the ROI. The objective function, given by the following equation, was set to maximize the sum of the electric field intensity component along the normal to the retinal surface in the ROI.

1 $$ \mathop \smallint \limits_{{{\Omega }_{{{\text{ROI}}}} }}^{{}} \left( {{\textbf{E}}\left( {\textbf{r}} \right) \cdot {\textbf{d}}\left( {\textbf{r}} \right)} \right){\text{~dr}} $$

Here **E(r)** and **d(r)** represent the electric field intensity vector and the unit vector perpendicular to the retinal surface at location **r**, respectively. ${\Omega}_{\text{ROI}}$ denotes the target ROI in the retina. The optimization problem is to determine the injection current **I**, where **I** represents the *L* × 1 sized injection current vector, with *L* being the number of active electrodes. Two constraints, C1 and C2, were introduced to consider the safety of the system.

2a $$\mathrm{C}1: {\Vert {\text{I}}\Vert }_{1}{ \leq 2}{\text{s}}_{\text{tot}}$$

2b $$\mathrm{C}2: {\Vert {\text{I}}\Vert }_{ \infty  }\leq {\text{s}}_{\text{ind}}$$

Here ${\Vert \cdot \Vert }_{1}$ and ${\Vert \cdot \Vert }_{ \infty  }$ are the L1-norm and the infinity norm, respectively. s_tot_ and s_ind_ represent the limit of the total injection current amplitude and the maximum current amplitude allowed for each active electrode, respectively^29^.

Before optimization, a coefficient matrix containing the relationship between the injection current and the electric field was calculated using the approach used in a previous study^30^. The electric field in the entire analysis domain was calculated using the FEM formulated with the electrostatic Laplace equation given by $-\nabla \bullet \left(\sigma \nabla V\right)=0$, where *V* is the electrical potential, and *σ* is the electrical conductivity. Dirichlet boundary conditions were then applied: + 1 V on the upper side of the active electrode and − 1 V on the upper side of the reference electrode. The calculated electric field was scaled such that the 1 mA of current was flowing into the reference electrode. We assumed that all tissue compartments were homogeneous, and their electrical conductivity values were set according to a previous study^18^. This process was repeated until the electric field distributions for each of the active electrodes were calculated. The electric field of the *m*-th element of the FE head model, **E**_***m***_, generated by the injection current vector, **I**, could be calculated using the superposition sum of the electric field vectors, pre-evaluated assuming the 1 mA current injection via a single active electrode and the reference electrode. Then, Eq. (1) can be rewritten as

3 $$  \mathop \smallint \limits_{{{\text{m~}} \in {\Omega }_{{{\text{ROI}}}} }}^{{}} \left( {{\textbf{E}}_{{\textbf{m}}} \left( {\textbf{r}} \right) \cdot {\textbf{d}}\left( {\textbf{r}} \right)} \right){\text{~dr}}$$

The injection currents for each active electrode that maximize the objective function in (3), considering constraints (2a) and (2b), were determined using CVX, a disciplined convex optimization solver package for Matlab^31^. The amplitude of the current flowing through the reference electrode was the summation of the injection currents of the individual active electrodes. Based on recent tES studies that reported that a total current of 1 mA injected with the conventional electrode montage did not evoke phosphene^16,32^, the limit of the total injection current (s_tot_) in (2a) and the maximum individual injection current amplitude (s_ind_) in (2b) were set to 1 mA and 0.5 mA, respectively. Additionally, another constraint (C3) was introduced to prevent a large amount of electric field from being delivered to the non-ROI regions.

4 $$\mathrm{C}3: {\text{E}}_{\text{max, outside}}{ \leq \alpha}$$

Here E_max, outside_ represents the maximum electric field formed outside the ROI on the retinal surface, and α denotes the threshold value for the electric field outside the ROI. It is well known that the stimulation focality and the mean electric field intensity in the ROI (referred to as E_mean_) have a tradeoff relationship^33^, where the focality can be defined as the ratio between E_max, outside,_ and E_mean_. As the value of α decreases, while the focality generally increases, the mean field intensity in the ROI decreases because the total injection current starts decreasing at a lower α value^33^. This study aimed to deliver the maximum electric field to the ROI and reduce the stimulation in the non-ROI regions, maintaining the total injection current at 1 mA. Accordingly, we empirically determined the value of α using an iterative CVX satisfying constraints C1, C2, and C3 by gradually reducing the value of α. After determining the optimal injection currents, the effectiveness of the proposed montage was validated by comparing it with three different conditions: the conventional montage, optimized suggested montage, and unoptimized suggested montage with equally distributed injection currents (injection current amplitude of 0.125 mA for one active electrode).

## Results

**Figure 3:**
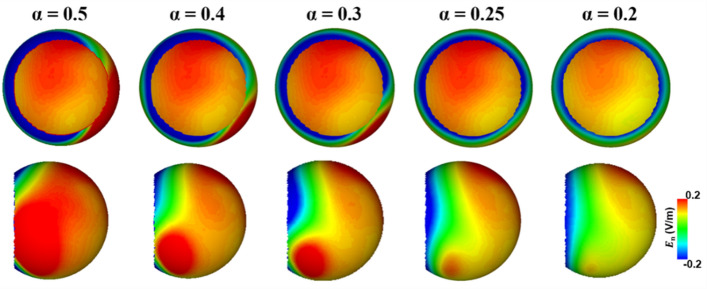
Electric field distributions in the retina based on the value of α within the constraint C3 for the iterative optimization.

**Figure 4:**
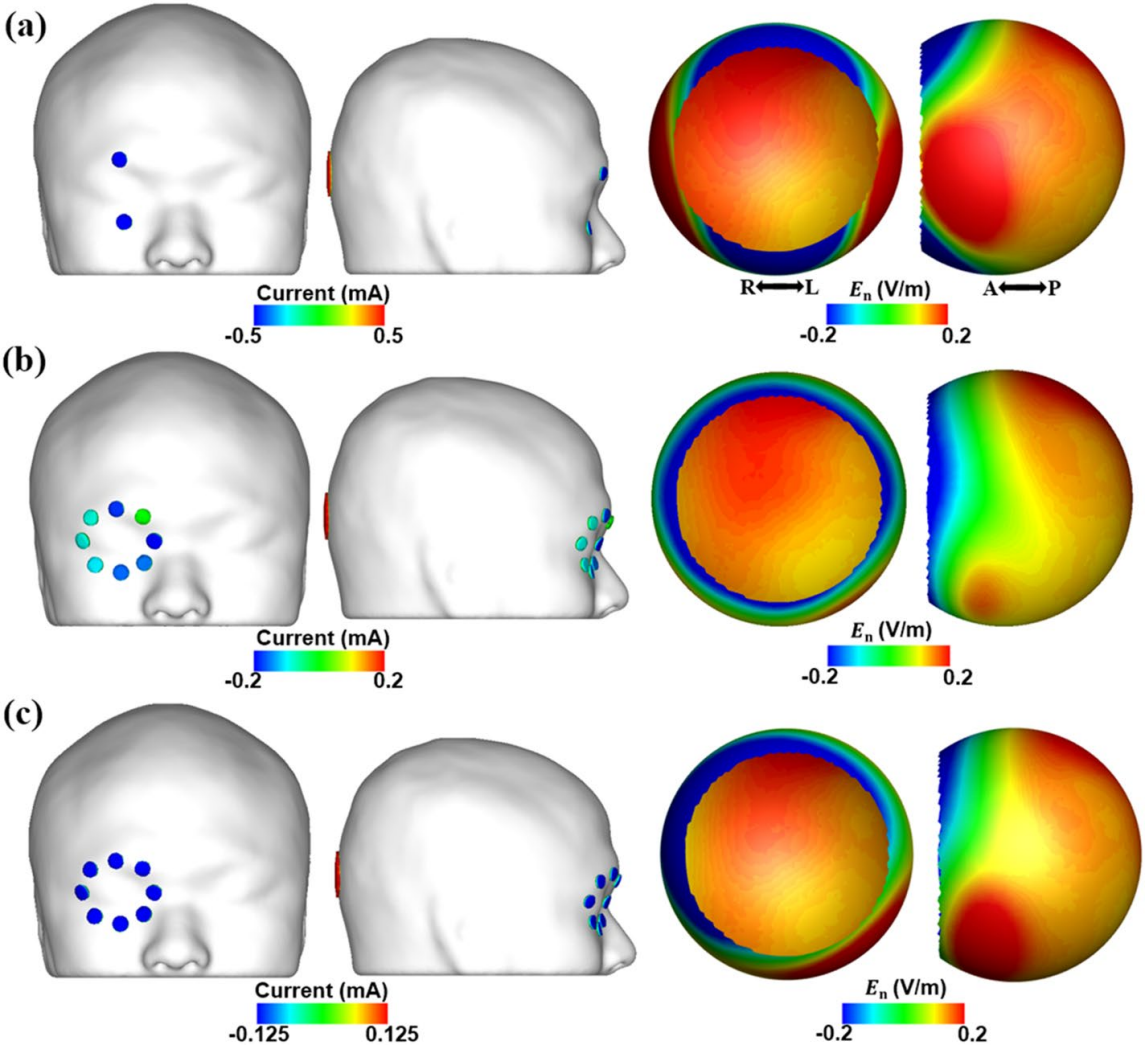
Injection current pattern and electric field distribution in the retinal surface when applying (**a**) conventional montage, (**b**) suggested montage with injecting the optimal injection currents, and (**c**) suggested montage with equally distributed injection currents. E_n_ represents the electric field in the direction normal to the retina surface.

It is important to note that we estimated the electric field in the direction normal to the surface of the retina, considering the direction of the arrangement of the RGC and photoreceptors, leading to both negative and positive electric field intensity values. Here, the negative values in the distribution represent the electric field coming out from the retinal surface. Figure 3 shows the electric field distribution on the retinal surface with respect to different threshold values of α for constraint C3. Table 1 lists the values of the maximum (E_max, ROI_) and mean electric fields in the ROI (E_mean, ROI_), the E_max, outside_, and the total injection current (I_total_), with respect to different α values. The results show that a smaller electric field was delivered to the regions outside the ROI as the value of α decreased. However, the electric field intensity delivered to the ROI also decreased when α fell below 0.25 V/m, with the total injection current being smaller than 1 mA. Accordingly, we set the value of α to 0.25 V/m, which led to maximum focality while the total injection current remained at 1 mA. Figure 4a–c show the electric field distributions on the retinal surface for the conventional montage, the suggested montage with optimized injection currents that set the value of α to 0.25 V/m, and the suggested montage with equally distributed injection currents, respectively. The amplitudes of the optimized injection currents of the suggested montage are listed in Table 3. Table 2 shows the maximum and mean electric field values in the retina under these conditions. Despite the injection of the same injection current of 1 mA, no comparable differences in the maximum and mean electric fields in the ROI were observed between the three conditions. However, while a much smaller maximum electric field of 0.25 V/m was delivered to the retina outside the ROI when applying the suggested montage, the maximum electric fields of 1.07 V/m and 0.44 V/m were delivered to the same region when applying the conventional montage and the suggested montage without the optimization, respectively. These results imply that the total injection current can be increased to deliver a much stronger electric field to the posterior retina without evoking phosphene owing to the stimulation of the anterior retina by employing the new multi-channel electrode montage with optimized injection currents, which would in turn increase the overall efficacy of tES.

**Table 1:** Maximum and mean electric field in the retina based on the value of α within constraint C3 for iterative optimization with the suggested montage.

	α = 0.5	α = 0.4	α = 0.3	α = 0.25	α = 0.2
E_max, ROI_	0.19 V/m	0.19 V/m	0.19 V/m	0.19 V/m	0.16 V/m
E_mean, ROI_	0.16 V/m	0.16 V/m	0.16 V/m	0.16 V/m	0.13 V/m
E_max, outside_	0.5 V/m	0.4 V/m	0.3 V/m	0.25 V/m	0.2 V/m
I_total_	1 mA	1 mA	1 mA	1 mA	0.86 mA

E_max, ROI_, E_mean, ROI_, E_max, outside_, I_total_ represent the maximum electric field in ROI, mean electric field in ROI, maximum electric field outside ROI, and total injection current, respectively.

**Table 2:** Maximum and mean electric field in the retina for three different conditions: conventional montage and suggested montage with and without optimizing injection currents.

	Conventional montage	Unoptimized suggested montage	Optimized suggested montage
E_max, ROI_	0.2	0.19	0.19
E_mean, ROI_	0.17	0.16	0.16
E_max, outside_	1.07	0.44	0.25

E_max, ROI_, E_mean, ROI_, E_max, outside_ represent the maximum electric field in ROI, mean electric field in ROI, and maximum electric field outside ROI, respectively.

(Unit: V/m).

## Discussions

tES, a noninvasive oculo-modulation method of improving visual functions, has attracted increased attention as a promising alternative to a relatively more invasive epiretinal implant or transcorneal electrical stimulation^1^. However, an electrode configuration, other than a conventional montage consisting of two active electrodes and a single reference electrode, to increase the therapeutic effect of tES is yet to be investigated. In this study, we propose a new tES electrode montage with eight active electrodes attached around the eye and a single reference electrode attached above the occipital area of the brain. We have optimized the injection current pattern that can effectively deliver a certain amount of electric field to the posterior retina while reducing the electric field being delivered to the anterior retina. For the proposed eight-channel montage, the optimized injection currents delivered a lower electric field to the anterior retina while preserving the electric field delivered to the posterior retina. This implies that the total injection current can be increased to deliver a stronger electric field to the posterior retina without evoking phosphene owing to the stimulation of the anterior retina.

**Figure 5:**
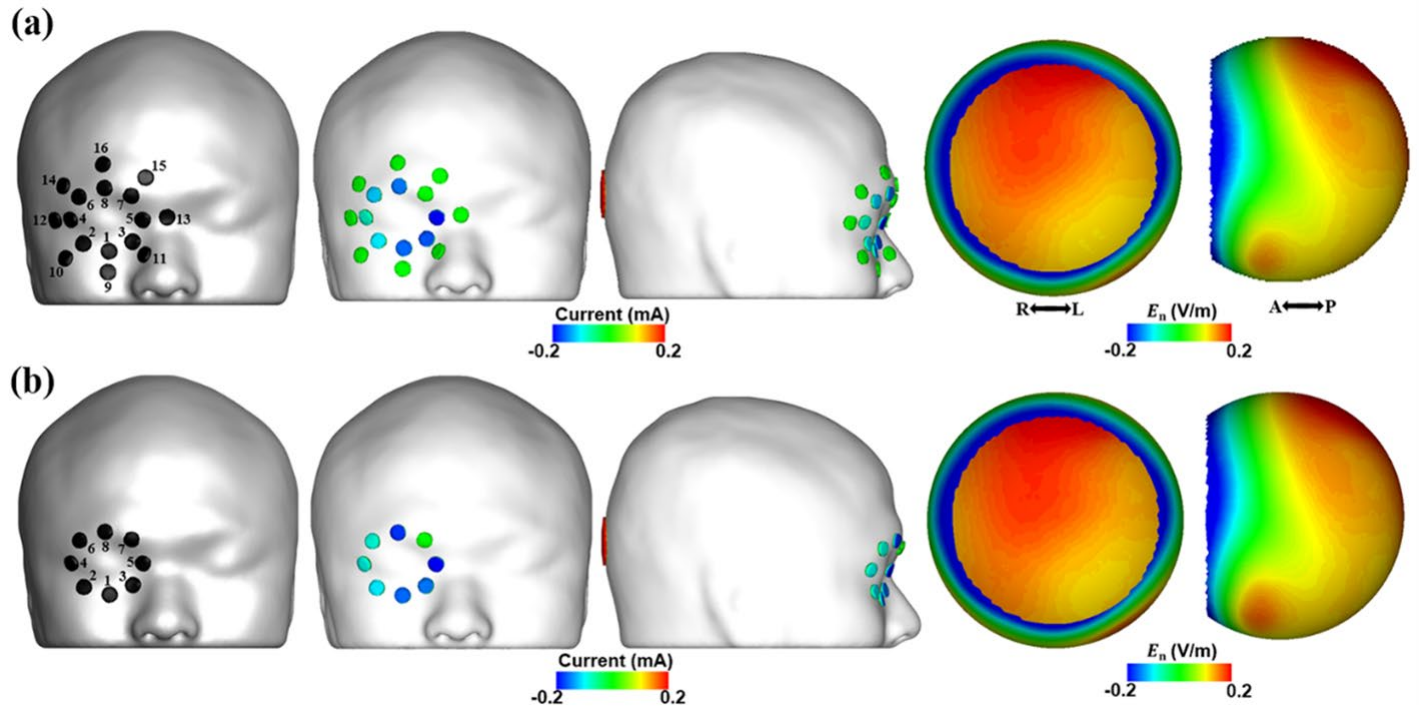
Injection current patterns and electric field distributions on the retinal surface when employing (**a**) electrode montage with 16 active electrodes, and (**b**) proposed electrode montage with eight active electrodes. The electrode numbers were marked next to the electrode locations. E_n_ represents the electric field component perpendicular to the retina surface.

Based on preliminary simulations, we determined the locations of eight active electrodes empirically. We first tried to employ 16 electrodes attached around the eye and one electrode attached over the occipital (see Fig. 5a). Figure 5 illustrates the injection current patterns and the electric field distributions in the retina for the electrode montages with 16 and eight active electrodes. Here, the maximum and mean electric fields in the ROI were 0.19 V/m and 0.16 V/m, respectively, identically for both electrode montages, and the maximum electric field outside the ROI was equal to 0.25 V/m, identically for both montages. The optimization results showed that there was no need to employ outer electrodes, thus, informing the decision to employ only eight active electrodes in further analyses. The amplitudes of the optimized injection currents for the suggested montage with 8 active electrodes and the montage with 16 active electrodes are listed in Table 3.

**Table 3:** Amplitudes of optimized injection currents for the suggested montage with 8 active electrodes and the montage with 16 active electrodes.

Electrode number	Suggested montage	Montage with 16 active electrodes
1 (9)	− 0.17	− 0.16 (0)
2 (10)	− 0.11	− 0.11 (0)
3 (11)	− 0.16	− 0.17 (0)
4 (12)	− 0.09	0.08 (0)
5 (13)	− 0.20	− 0.21 (0)
6 (14)	− 0.09	− 0.12 (0)
7 (15)	0	0 (0)
8 (16)	− 0.18	− 0.15 (0)
Return	1	1

(Unit: mA).

In case of the montage with 16 active electrodes, the injection current amplitudes for the electrode numbers 9 to 16 are given in the parentheses.

**Figure 6:**
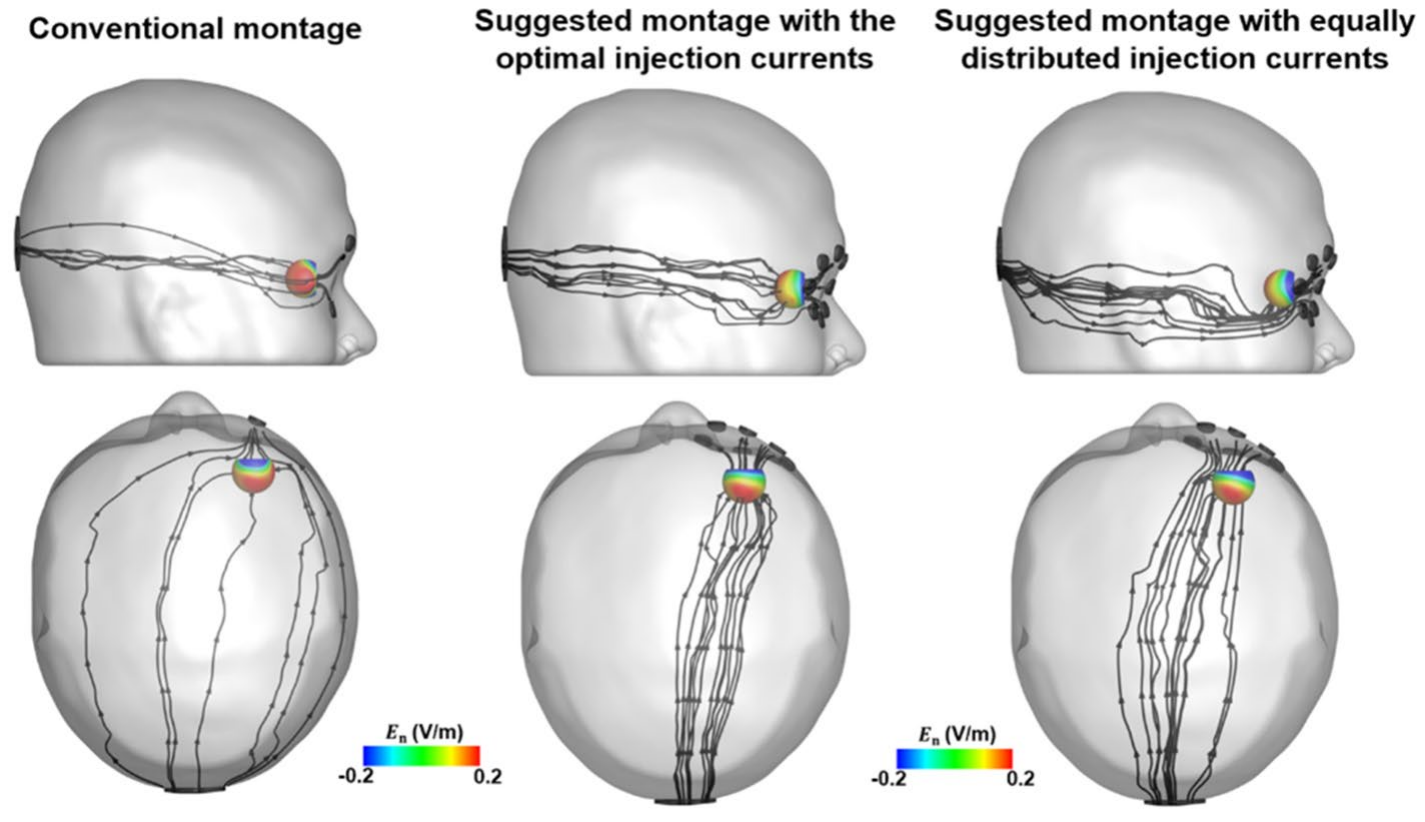
Illustration of streamlines and the distributions of electric fields on the retinal surface for three different conditions (conventional montage, suggested montage with the optimal injection currents, and suggested montage with equally distributed injection currents).

Additionally, we drew streamlines of electric fields to explore how the resultant electric fields were formed with respect to three different electrode montage conditions (see Fig. 6). The results for the conventional electrode montage revealed that most of the electric fields flowed through the tissue next to the eyeball, with some electric fields delivered to the peripheral side of the retina simultaneously. This resulted in a considerably high electric field in the regions outside the ROI. However, the suggested montage, with the injection of the optimal current, provided a more concentrated delivery of the electric field into the posterior retina compared to the conventional montage. Thus, a relatively small electric field was formed in regions outside the ROI. When it was assumed that equal currents flowed into all eight electrodes, however, electric fields preferentially flowed under and to the left of the retina rather than through the center of the posterior retina. Our results indicate the possibility to steer electric fields to be delivered to the desired target regions, avoiding the circumscribed regions being stimulated by the optimization of injection currents. Consequently, using multi-channel tES with the optimized current pattern could prevent the electric fields from flowing into tissues other than in the posterior retina.

As aforementioned, the therapeutic effect of tES results from the entrainment of rhythmic firing of the RGCs by tES^34^, which is possible even when a relatively small electric field is delivered to RGCs^12^. Indeed, it is known that alternating electric fields whose amplitude range from 0.1 to 0.2 V/m was sufficient to entrain endogenous neural oscillations^35–38^. Therefore, it is believed that the mean electric field of 0.16 V/m delivered to the posterior retina might have a sufficient possibility to induce rhythmic firing of the RGCs. Furthermore, there is still room for increasing the total injection current in the proposed electrode montage because the maximum electric field outside ROI (posterior retina) in the conventional montage was approximately four times larger than that in the proposed montage, as presented in Table 2.

For human trials with conventional montage, the injection current is determined based on the phosphene threshold that induces retinal phosphene^12^. As the electric field outside the ROI is four times larger than that in the ROI for the conventional montage, the phosphene threshold should be determined by the excitation of RGCs outside the ROI. Therefore, a sufficient stimulating current could not be delivered to the posterior retina, and the efficacy of the therapy was consequently degraded. A clinical tES study with RP patients demonstrated that using a stronger injection current than the individual phosphene threshold showed therapeutic effects, while the injection current determined based on the individual phosphene threshold did not exhibit any improvement in visual functions^16^. Schatz et al. reported that the visual field of RP patients was improved or remained unchanged by injecting the current equivalent to 150% of individual phosphene thresholds, whereas there was no marked tendency when injecting the current equivalent to 66% of individual phosphene threshold during transcorneal electrical stimulation^4^. Although the relationship between the strength of the injection current and the therapeutic effects of tES should be further investigated through a series of human trials, it seems evident that retinal stimulation with a stronger electric field allows for the elevation of the therapeutic effects of tES. Based on our simulation results, we strongly believe that the proposed montage should provide an opportunity to increase the total injection current while reducing the possibility of retinal phosphene, which would allow for better treatment opportunities for patients.

For the practical use of the suggested montage, safety issue needs to be addressed. According to a previous study on the safety of transcranial electrical stimulation, injection of 3 mA current through electrodes with a diameter of 0.8 cm did not cause any side effects, including skin burn, skin redness, and pain^39^. It is to be noted that the maximum current amplitude injected through each active electrode was just 0.2 mA in our simulations. As the larger sized electrodes with a diameter of 1 cm were assumed in this study, we strongly believe that it would be safe to inject current much smaller than 3 mA in the proposed montage. In addition, a previous study reported that the maximal electric field amplitude that does not cause any tissue damage is 42 V/m^18^. As much smaller electric field is delivered to the retinal tissues in the suggested montage, the use of the suggested montage will not cause any damage to the retinal tissue. Nevertheless, these safety issues need to be further addressed through in-vivo experiments in the future studies.

**Figure 7:**
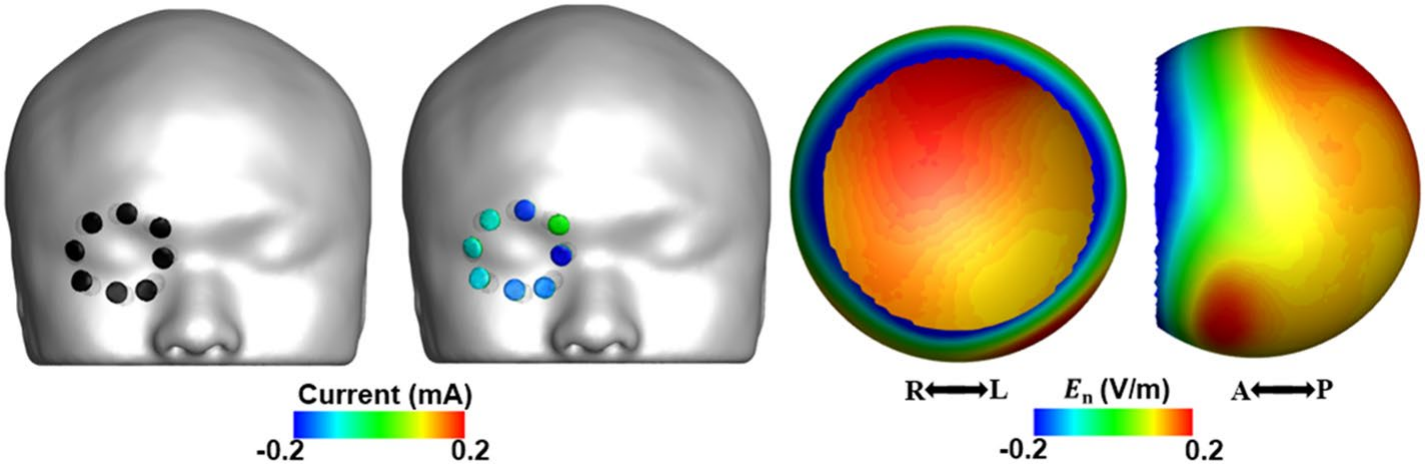
Injection current pattern and electric field distribution in the retinal surface when the active electrodes were slightly displaced from their original locations in the clockwise direction.

In the practical application of the proposed multi-channel tES, the locations active electrodes can be slightly different from those assumed in the numerical simulations. To investigate the influence of slight electrode displacement on the resultant electric field distributions, we performed an additional simulation. We rotated eight active electrodes in the clockwise direction, as shown in Fig. 7, when the displacement of each electrode was assumed to be a half of the electrode diameter (0.5 cm), which is thought to be a sufficiently large displacement. Compared to the original field analysis results provided in Fig. 4b and Table 2, only small difference was observed in both the overall field distribution and the electric field quantities. E_max_, E_mean_, and E_max, outside_ values of the newly calculated field distribution were 0.18, 0.15, and 0.27, respectively, and those of the original distribution were 0.19, 0.16, and 0.25, respectively. These results suggest that slight displacements of active electrodes occurring during electrode attachment would not significantly influence the overall electric field distributions. Based on a previous study, the eye rotates with a rotation angle of approximately 10° during eyelid closure^40^. To demonstrate the effect of eye rotation, we additionally calculated the electric field distributions assuming that the eye was rotated either horizontally or vertically with a 10° rotation angle. For each simulation, the optimal injection currents obtained assuming no eye rotation was applied. Our simulation results showed that there was no significant change in the overall electric field distributions in the retina after rotating the eye (see Fig. S1 and Table S1 in Supplementary Materials), although the portion of retina being subjected to the maximum electric field changed with rotation. Even though E_max, outside_ slightly increased due to the eye rotation, the value was still much smaller than that in the conventional tES montage. Our results suggest that the eye rotation during eyelid closure would not significantly influence the overall electric fields when the suggested electrode montage is employed.

In the present study, we attached the reference electrode to the occipital pole based on previous studies^10^. If the reference electrode is attached to a different area, the current pattern at each electrode might change as the location of the return or the reference electrodes influences the formation of electric fields^41^. As per our findings, we believe that the suggested montage with the optimal injection currents might produce the maximal focality for targeting the posterior retina regardless of the slight changes in the reference electrode’s location. However, there might be a possibility of improving the focal delivery of electric fields to the target by attaching the reference electrode to other areas, such as the neck and the arm, as considered in previous studies^12,18^. Thus, it would be an interesting topic for future research to employ a full human body model to verify the effectiveness of tES with the reference electrode attached to extra-cephalic regions.

**Figure 8:**
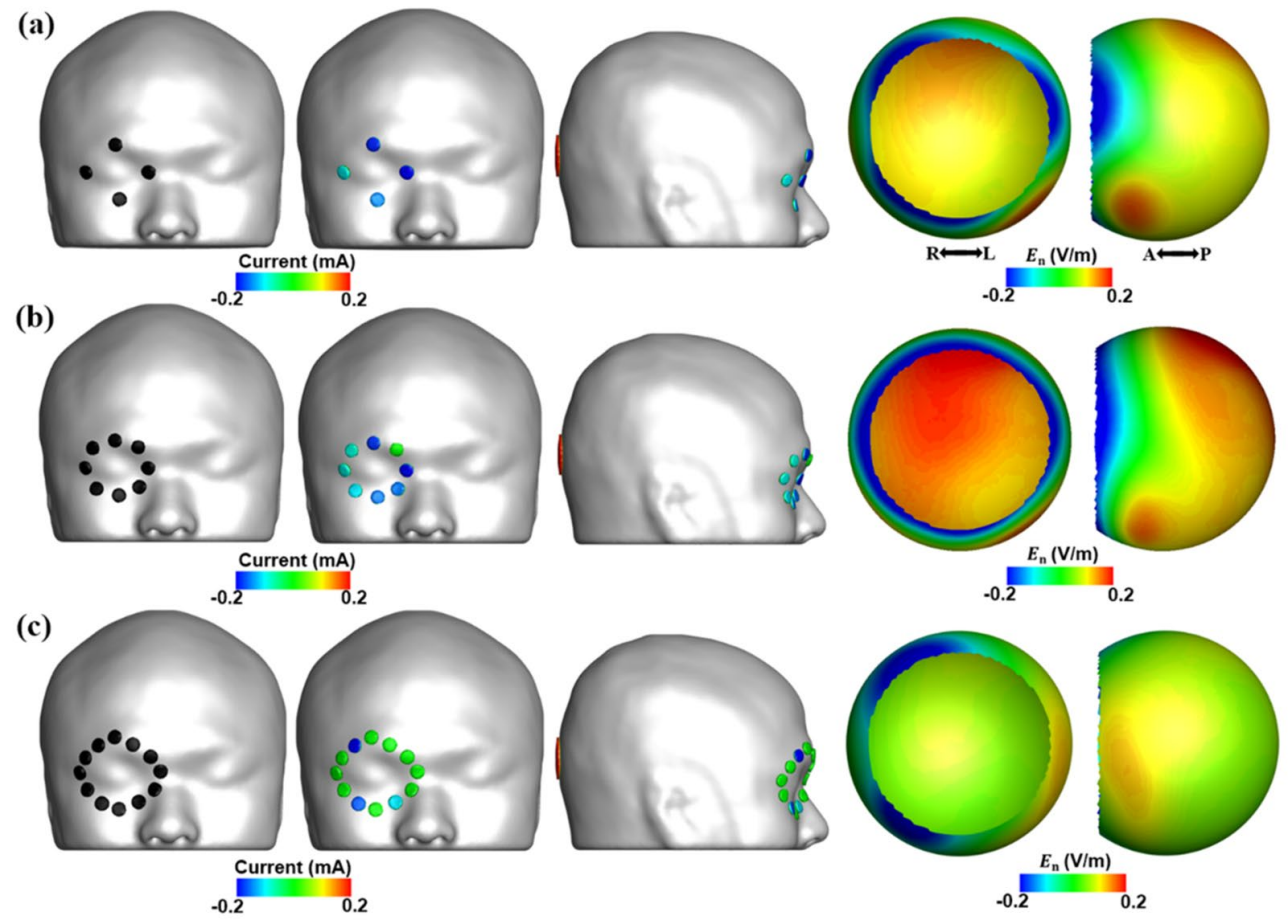
Injection current pattern and electric field distribution in the retinal surface when applying (**a**) an electrode montage with 4 active electrodes, (**b**) the suggested electrode montage with eight active electrodes, and (**c**) an electrode montage with 12 active electrodes. For each montage, optimal injection currents were found from the optimization procedure. E_n_ represents the electric field in the direction normal to the retina surface.

To demonstrate the influence of the number of active electrodes on the effectiveness of multi-channel tES, additional simulations were conducted with different electrode montages with four and 12 active electrodes, when the same optimization process used in this study was employed. For the montage with 12 active electrodes, the active electrodes were attached approximately 2.7 cm away from the center of the eye, to keep the minimum distance between adjacent electrodes larger than 0.5 cm for preventing electrical short circuit in practical applications. It is to be noted that in the cases of the montages with four and eight active electrodes, the distance between the active electrodes and the center of the eye was assumed to be approximately 2 cm. After the optimization procedure, we compared electric field distributions in the retina obtained using three different montages with four, eight, and 12 active electrodes. The simulation results showed that the suggested montage with eight active electrodes was more beneficial to the focal stimulation of the posterior retina than the montages with four and 12 active electrodes, as presented in Fig. 8 and Table 4. When the montages with four and eight active electrodes were compared, it was found that the use of more active electrodes allows for more focalized stimulation of the posterior retina. However, when the electrodes were attached slightly farther from the eye, considerable reduction of electric field in the posterior retina was observed, which could not be easily overcome by simply using more numbers of electrodes. This finding is consistent with our original findings showing that no current was actually flowing through outer electrodes in the montage with 16 active electrodes (see Fig. 5). Based on these simulations, it could be confirmed that the suggested montage with eight active electrodes allows for more effective delivery of the electric fields to the posterior retina than the other montages.

**Table 4:** Maximum and mean electric field in the retina for three different conditions: (1) a montage with four active electrodes, (2) the suggested montage with eight active electrodes, and (3) a montage with 12 active electrodes.

	Montage with four active electrodes	Suggested montage with eight active electrodes	Montage with 12 active electrodes
E_max, ROI_	0.14	0.19	0.07
E_mean, ROI_	0.11	0.16	0.06
E_max, outside_	0.25	0.25	0.25

E_max, ROI_, E_mean, ROI_, E_max, outside_ represent the maximum electric field in ROI, mean electric field in ROI, and maximum electric field outside ROI, respectively.

(Unit: V/m).

In addition, our simulation results were not validated via in vivo or ex vivo experiments. Although the optimization and simulation methods employed in this study have already been widely employed for the focal stimulation of cortical regions in transcranial electrical stimulation^29,30,42–45^, it would be a promising future topic to investigate the distribution of the electric fields in the retina in in vivo experimental conditions and quantitatively compare the difference between the computed and measured electric fields.

## Conclusions

This study suggests a novel electrode montage with multiple electrodes attached around the eye to stimulate the posterior retina, where retinal cells are densely distributed, for the first time. Our suggested montage, with the optimization of the injection current pattern, allows for more focal stimulation of the posterior retina while preventing the electric field from being delivered to other regions in the retina more effectively than the conventional montage and the same multi-electrode montage without the optimization of injection currents. Our simulation results demonstrated that the total injection current can be increased to deliver a much stronger electric field to the posterior retina without evoking retinal phosphene owing to the stimulation of the anterior retina by employing the new multi-channel electrode montage with optimized injection currents. Although additional in-vivo experimental studies are necessary, the proposed approach is thought to have a potential to become a better treatment option for patients who do not show therapeutic effects of tES.

## Supplementary Information

Supplementary Information 1.

## Data Availability

Please contact the corresponding author (ich@hanyang.ac.kr) for data requests.
