# Supplementary material for: Multi-channel transorbital electrical stimulation for effective stimulation of posterior retina
Source: Sci Rep. 2021 May 7;11:9745. doi: 10.1038/s41598-021-89243-y (PMC8105361; doi:10.1038/s41598-021-89243-y)
Supplement: Supplementary file 1 — Supplementary Information 1. [file 41598_2021_89243_MOESM1_ESM.pdf]

Supplementary materials

Multi-Channel Transorbital Electrical Stimulation for Effective Stimulation of Posterior Retina

Sangjun Lee[1], Jimin Park[1], Jinuk Kwon[1], Dong Hwan Kim[2], and Chang-Hwan Im[1, 3, 4*]

[1] Department of Electronic Engineering, Hanyang University, Seoul, Republic of Korea

[2] Center for Intelligent & Interactive Robotics, Korea Institute of Science and Technology, Seoul, Republic of Korea

[3] Department of Biomedical Engineering, Hanyang University, Seoul, Republic of Korea

[4] Department of HY-KIST Bioconvergence, Hanyang University, Seoul, Republic of Korea

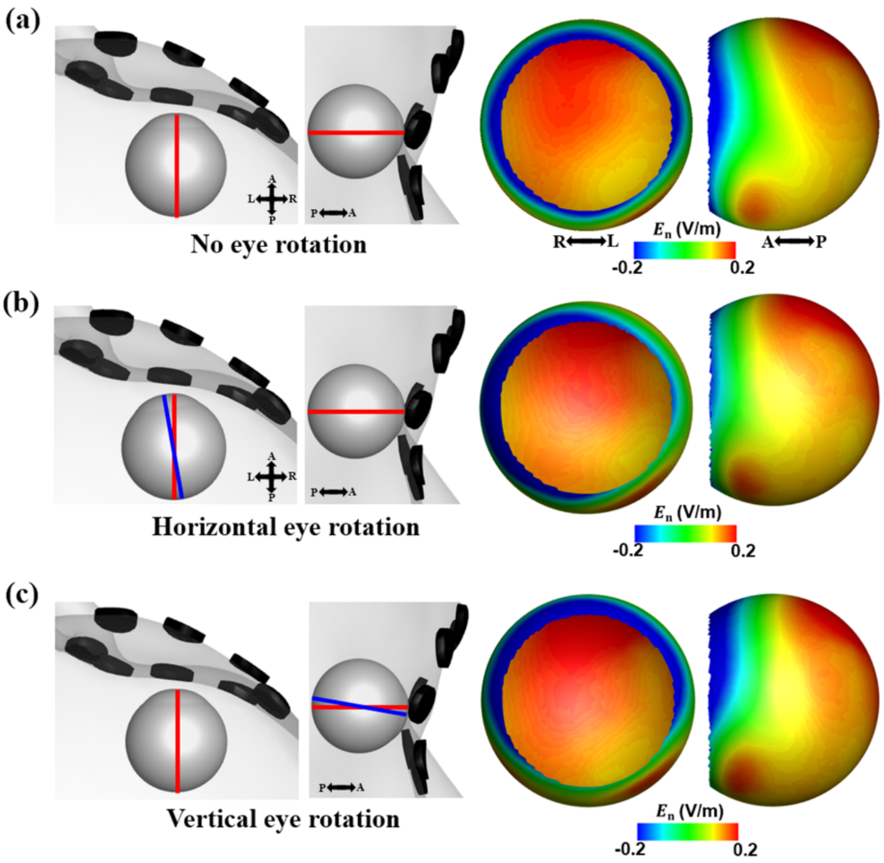

Figure S1. Illustrations of the eye conditions and electric field distributions in the retinal surface when there is (a) no eye rotation, (b) horizontal eye rotation with an angle of 10 degrees, and (c) vertical eye rotation with an angle of 10 degrees. For each condition, the same optimal injection currents obtained from the suggested montage assuming no eye rotation were applied. E_n represents the electric field in the direction normal to the retina surface.

Table S1. Maximum and mean electric field in the retina for three different conditions: (1) no eye rotation, (2) horizontal eye rotation with an angle of 10 degrees, and (3) vertical eye rotation with an angle of 10 degrees. $E_{max, ROI}$, $E_{mean, ROI}$, $E_{max, outside}$ represent the maximum electric field in ROI, mean electric field in ROI, and maximum electric field outside ROI, respectively.

	**No eye rotation**	**Horizontal eye rotation**	**Vertical eye rotation**
$E_{max, ROI}$	0.19	0.20	0.20
$E_{mean, ROI}$	0.16	0.17	0.18
$E_{max, outside}$	0.25	0.32	0.34

(Unit: V/m)
